# Supplementary material for: Single-stranded DNA drives σ subunit loading onto mycobacterial RNA polymerase to unlock initiation-competent conformations
Source: Nucleic Acids Res. 2025 Apr 16;53(7):gkaf272. doi: 10.1093/nar/gkaf272 (PMC12000874; doi:10.1093/nar/gkaf272)
Supplement: gkaf272_Supplemental_Files [file gkaf272_supplemental_files.zip › Vishwakarma_etal_SI_figures_tables_300dpi_R2.pdf]

Supplementary Information

Single-stranded DNA drives σ subunit loading onto mycobacterial RNA polymerase to unlock initiation-competent conformations

Rishi Kishore Vishwakarma, Nils Marechal, Zakia Morichaud, Mickael Blaise, Emmanuel Margeat, Konstantin Brodolin

Supplementary Table S1. DNA oligonucleotides

Oligo name	Sequence	Label	Experiment
-10 ssDNA	5’-TGCGTATAATGTGTGGA-3’	-	Cryo-EM, smFRET
-10 ssDNA	5’-[Cyanin5]TGCGTATAATGTGTGGA-3’	Cy5	DNA-protein cross-linking
-10 ssDNA	5’-[6FAM]TGCGTATAATGTGTGGA-3’	Fluorescein	MDS
-10 ssDNA truncated	5’-TATAATGTGTGGA[Cyanin5]-3’	Cy5	DNA-protein cross-linking
-11C ssDNA	5’-TGCGTCTAATGTGTGGA-3’	-	smFRET
-11C ssDNA	5’-[6FAM]TGCGTCTAATGTGTGGA-3’	Fluorescein	MDS
*SigA*P us-fork top strand	5'-CGCTCGGGCTGTACTCGTGCGCAGTAAAGTTACAATGGTC-3’	-	EM
*SigA*P us-fork bottom strand	5'-AACTTTACTGCGCACGAGTACAGCCCGAGCG-3’	-	EM
RbpAR88R89A forward primer	5'-GGGACATGCTGCTGGAGGCCGCTTCCATCGAAGAACTCG-3'	-	site-directed mutagenesis of *rv2050*
RbpAR88R89A Reverse primer	5'-CGAGTTCTTCGATGGAAGCGGCCTCCAGCAGCATGTCCC-3	-	site-directed mutagenesis of *rv2050*

Supplementary Table S2 cryo-EM data collection, refinement and validation statistics

	Consensus-I	Consensus-II	σR4-docked	σR4-undocked	Clamp swiveled	Clamp unswiveled
**Data collection**						
Pixel size (Å)	0.862					
Voltage (kV)	300					
Electron dose (e^{-1} Å $^{-2}$)	55.735					
Defocus range (µm)	-0.8 - -2.5					
**Reconstruction**						
Particles used in reconstruction	290,345	167,825	72,799	67,957	21,873	36,319
Map resolution (Å) FSC threshold 0.143	3.19	3.33	3.43	3.48	4.33	3.79
**Refinement**						
Resolution FSC threshold 0.5	3.4	-	3.7	3.7	-	-
Map CC (whole map) (volume)	0.81	-	0.82	0.79	-	-
Map CC (peaks)	0.74	-	0.79	0.76	-	-
**RMSD**						
Bond length (Å)	0.004	-	0.004	0.004	-	-
Bond angle (°)	0.931	-	0.959	0.929	-	-
**Ramachandran Plot**						
Preferred regions (%)	97.23	-	97.08	97.02	-	-
Allowed regions (%)	2.77	-	2.92	2.98	-	-
Outliers (%)	0.00	-	0.00	0.00	-	-
**Validation**						
MolProbity score	1.21	-	1.47	1.38	-	-
All-atom clashscore	2.8	-	5.67	4.31	-	-
Rotamer outliers (%)	0.74	-	0.18	0.95	-	-
EM accession	EMD-50508	EMD-50509	EMD-50510	EMD-50511	EMD-50512	EMD-50514
PDB accession	9FJP	-	9FJR	9FJS	-	-

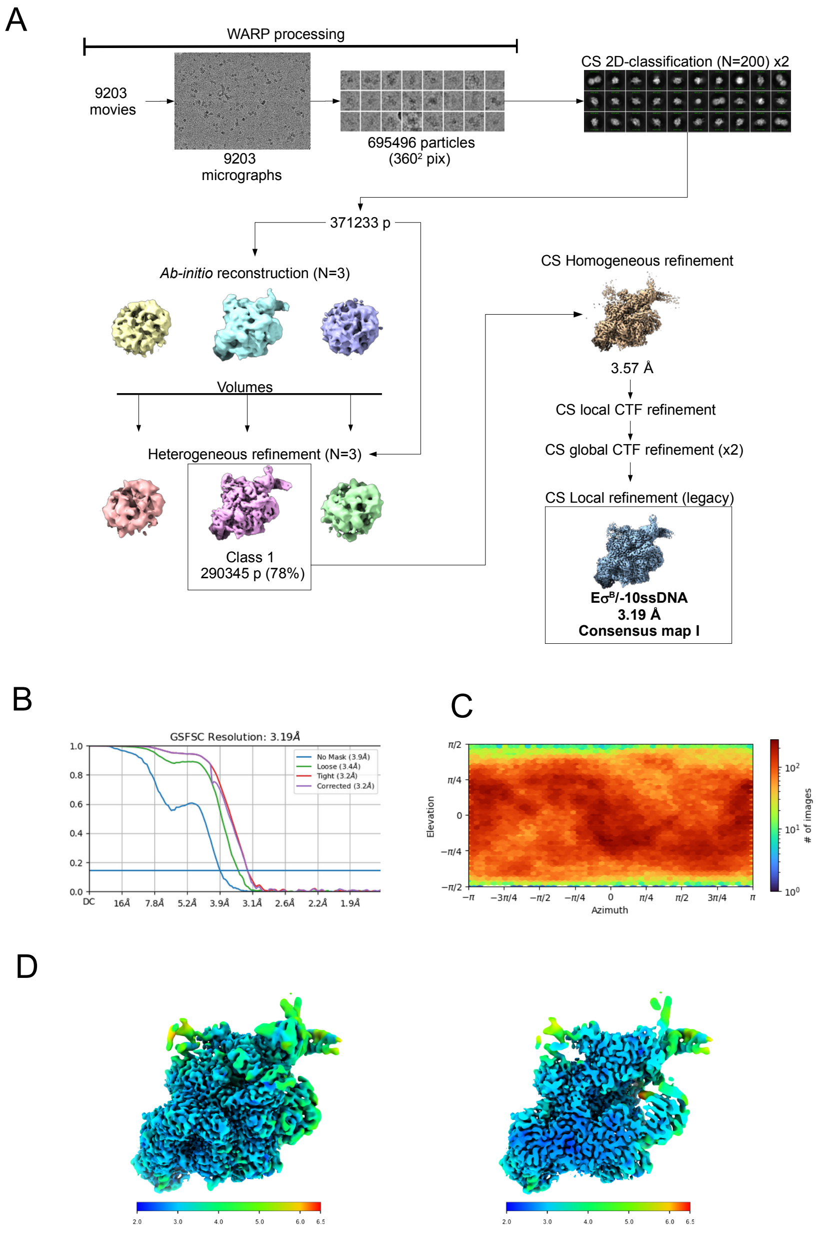

Figure S1. Reconstruction and characterization of the consensus-I map.
(A) CryoSPARC pipeline for consensus-I map. (B) Gold-standard FSC calculated for the map in cryoSPARC v3.3.2. The dotted line shows the 0.143 FSC cutoff. (C) Angular distributions for particles projections calculated in cryoSPARC and presented as a heat map. (D) Cryo-EM density map and sliced map (on the right) colored according to the local resolution calculated at 0.143 FSC.

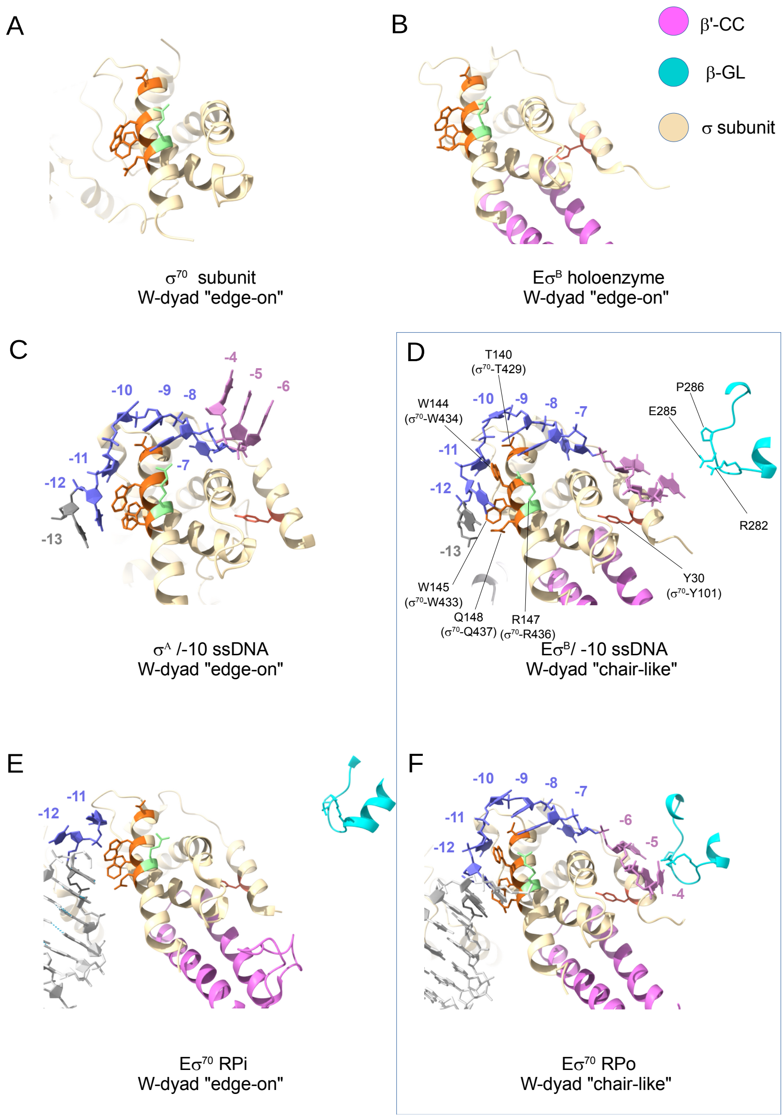

Figure S2 Architecture of the σ subunit ssDNA-binding interface at different steps of transcription initiation.

(A) Structure of the *E. coli* σ^{70} fragment (PDB:1SIG). (B) Structure of the Mtb $E\sigma^{B}$ holoenzyme (PDB:7PP4). (C) Structure of the *T. aquaticus* σ^{A}/-10 ssDNA complex (PDB: 3UGP). (D) Structure of the E σ^{B}/-10 ssDNA complex (current study). (E) Structure of the *E. coli* $E\sigma^{70}$ RPi (PDB: 6PSR) (F) Structure of the *E. coli* $E\sigma^{70}$ RPo (PDB: 7MKD). Ribbon models of the σ subunit colored wheat, β' subunit clamp helices (β'-CH) in pink, β subunit gate loop (β-GL) in cyan. The key residues of σ implicated in -10 recognition and isomerization of RPc to RPo are shows as stick molecular model colored in orange: W-dyad, Q148 (σ^{70}-Q437, (Waldburger *et al*, 1990), T140 (σ^{70}-T429) critical for promoter melting (Schroeder *et al*, 2008; Waldburger & Susskind, 1994) and green R147 (σ^{70}-R436, (Fenton *et al*, 2000). Y30 (σ^{70}-Y101) is essential for stimulation of the -10 binding by the RNAP core (Zenkin *et al*, 2007). The conserved residues of β-GL implicated in RPo formation are show as stick molecular model.

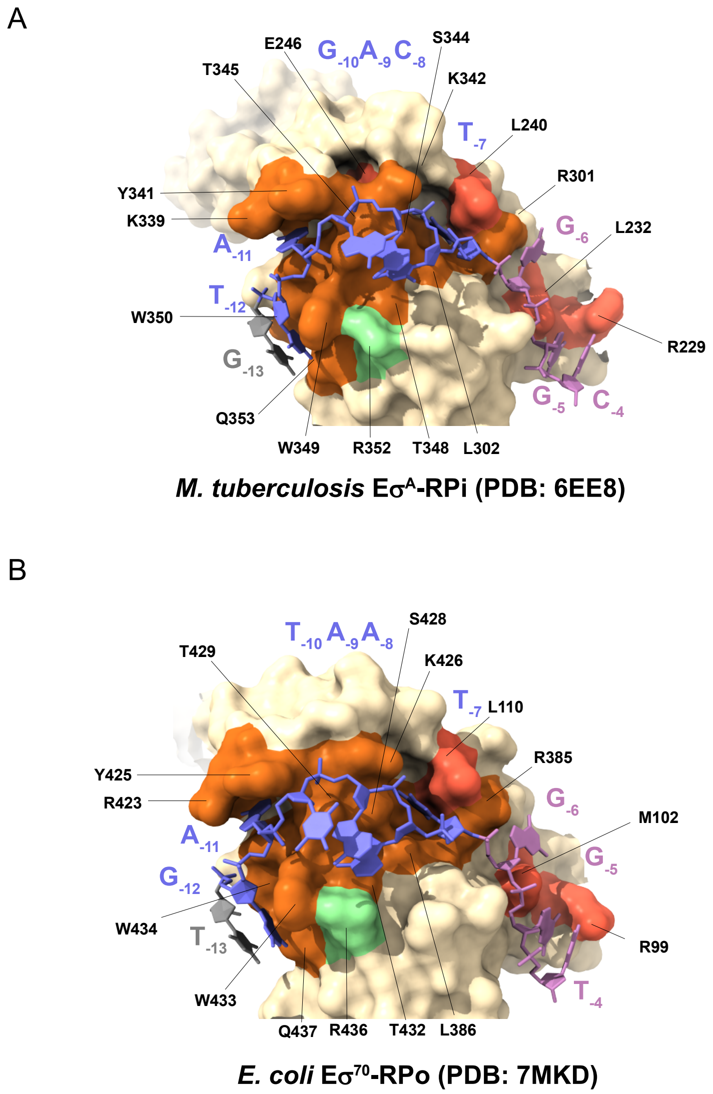

Figure S3. Architecture of the σ/-10 ssDNA interactions in *Mtb* RPi and *E. coli* RPo

Comparison of the of the σ/ssDNA binding interfaces in RPo formed by *Mtb*Eσ^{A} (panel A, PDB: 8EE8) and *E. coli* Eσ^{70} (panel B, PDB: 7MKD). The σ domain 2 is shown as molecular surface colored in wheat. Residues interacting with ssDNA are colored in orange (σ region 2) and tomato (σ region 1.2). σ^{A}-R352 and σ^{70}-R436 making holoenzyme-specific π-interactions with W-dyad is colored light green. ssDNA is show as stick molecular model with filled sugars and bases. Color codes as in Figure 3B.

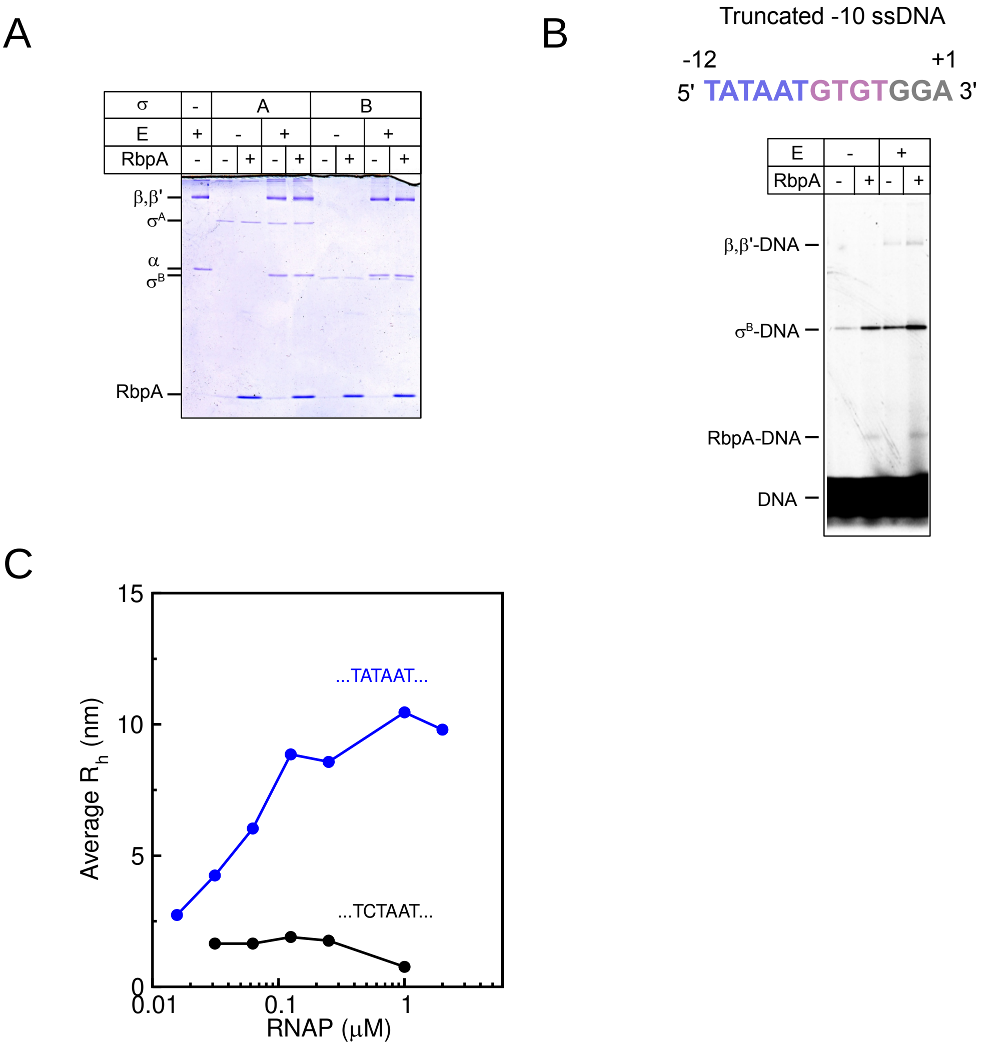

Figure S4. Analysis of the interactions between -10 ssDNA and σ subunits
(A) Probing of the DNA-protein interactions by formaldehyde cross-linking. Indicated combinations of MtbRNAP core, σ^A, σ^B and RbpA were cross-linked to fluorescent -10 ssDNA and resolved on SDS-PAGE. The Coomassie blue stain is shown. (B) Probing of the DNA-protein interactions by formaldehyde cross-linking. Indicated combinations of MtbRNAP core (E), σ^B and RbpA were cross-linked to a truncated version of fluorescent -10 ssDNA (shown on the top) and resolved on SDS-PAGE. (C) Measurement of -10 ssDNA (blue circles) and -11C ssDNA (black circles) binding to $E\sigma^B$ by MDS. Graph shows average R_h as function of protein concentration.

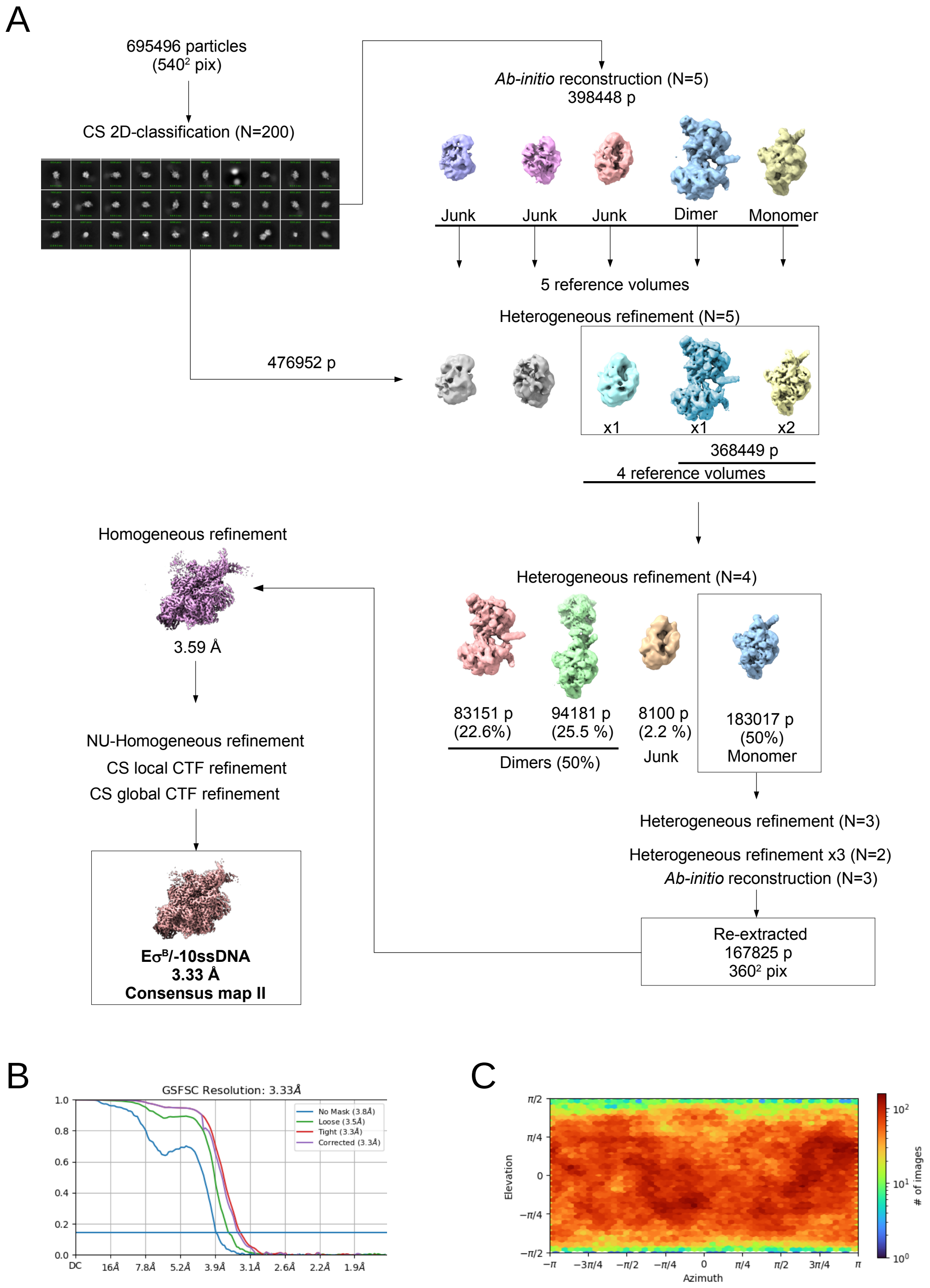

Figure S5. Reconstruction and characterization of the consensus-II map.
(A) cryoSPARC pipeline for consensus II map (B) Gold-standard FSC calculated for the map in cryoSPARC v3.3.2. The dotted line shows the 0.143 FSC cutoff. (C) Angular distributions for particles projections calculated in cryoSPARC and presented as a heat map.

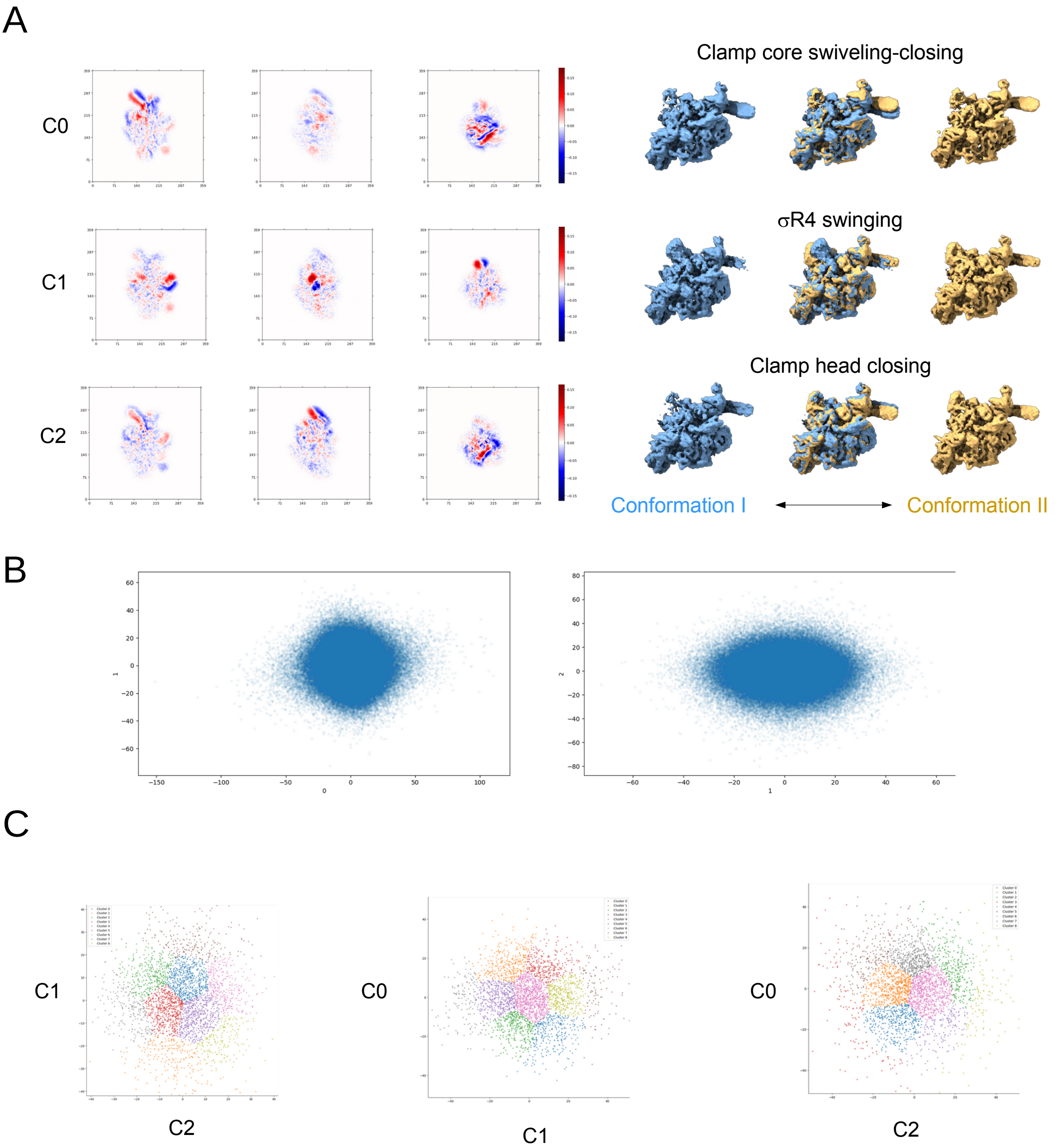

Figure S6. Conformational heterogeneity of the Eσ^B/-10 ssDNA complex explored by the 3D variability analysis (3DVA) in cryoSPARC

(A) On the left: slices in x-y for three subspace directions of each reaction coordinate, C0, C1 and C3. The slices show positive (red) and negative (blue) values. On the right: 3D density maps generated in 3DVA along each variability component. Two utmost representative conformations are shown . (B) 2D-scatter plots of particles coordinates distribution over 3 components. (C) 3DVA cluster analysis of the particles latent coordinates to reveal cross-correlation between variability over each reaction coordinate.

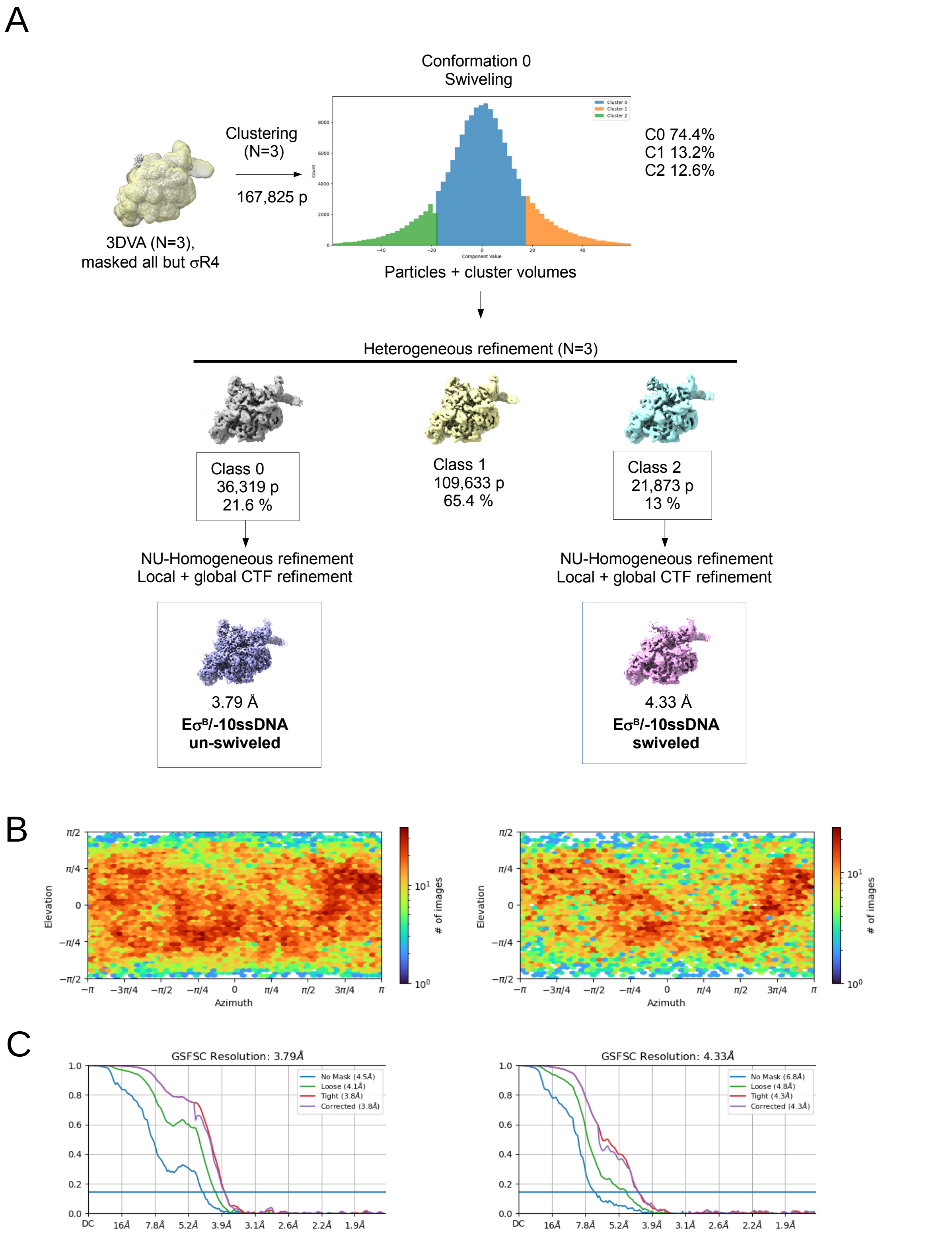

Figure S7. Separation of the clamp conformations.
(A) cryoSPARC pipeline for conformation 0 (left) and conformation 2 (right). 3DVA analysis was performed with particles from the consensus II map refinement job with the mask on the RNAP excluding σ subunit domain 4. (B) Angular distributions for particles projections calculated in cryoSPARC and presented as a heat map. (C) Gold-standard FSC calculated for the map in cryoSPARC v3.3.2. The dotted line shows the 0.143 FSC cutoff.

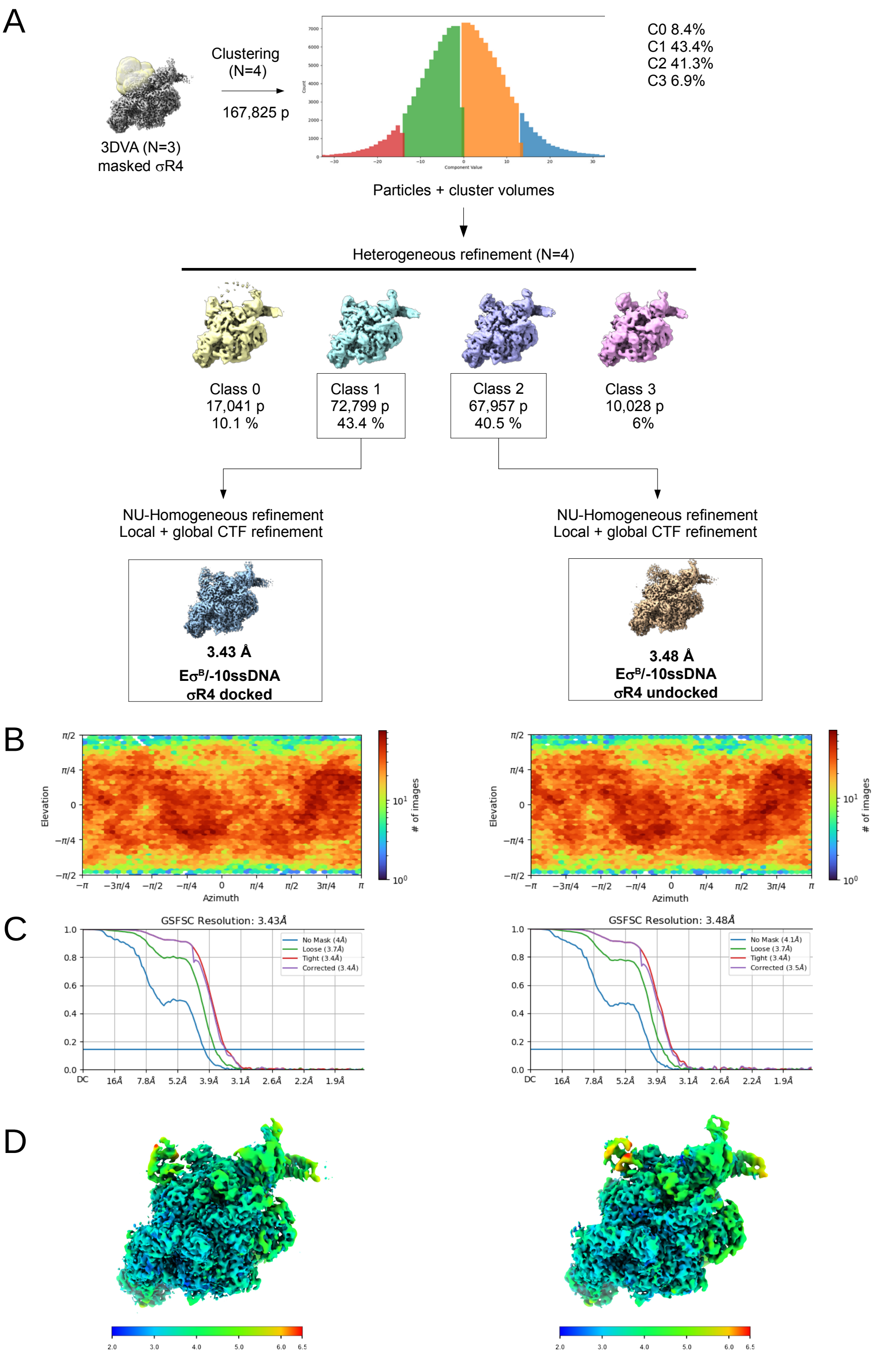

Figure S8 Separation of the docked and undocked conformations of σR4.
(A) cryoSPARC pipeline for docked and undocked σR4 RNAPs. 3DVA analysis was performed with particles from the consensus II map refinement job with the mask on the σ subunit region 4. (B) Angular distributions for particles projections calculated in cryoSPARC and presented as a heat map. (C) Gold-standard FSC calculated for the map in cryoSPARC v3.3.2. The dotted line shows the 0.143 FSC cutoff. (D) Cryo-EM density maps colored according to the local resolution calculated at 0.143 FSC.

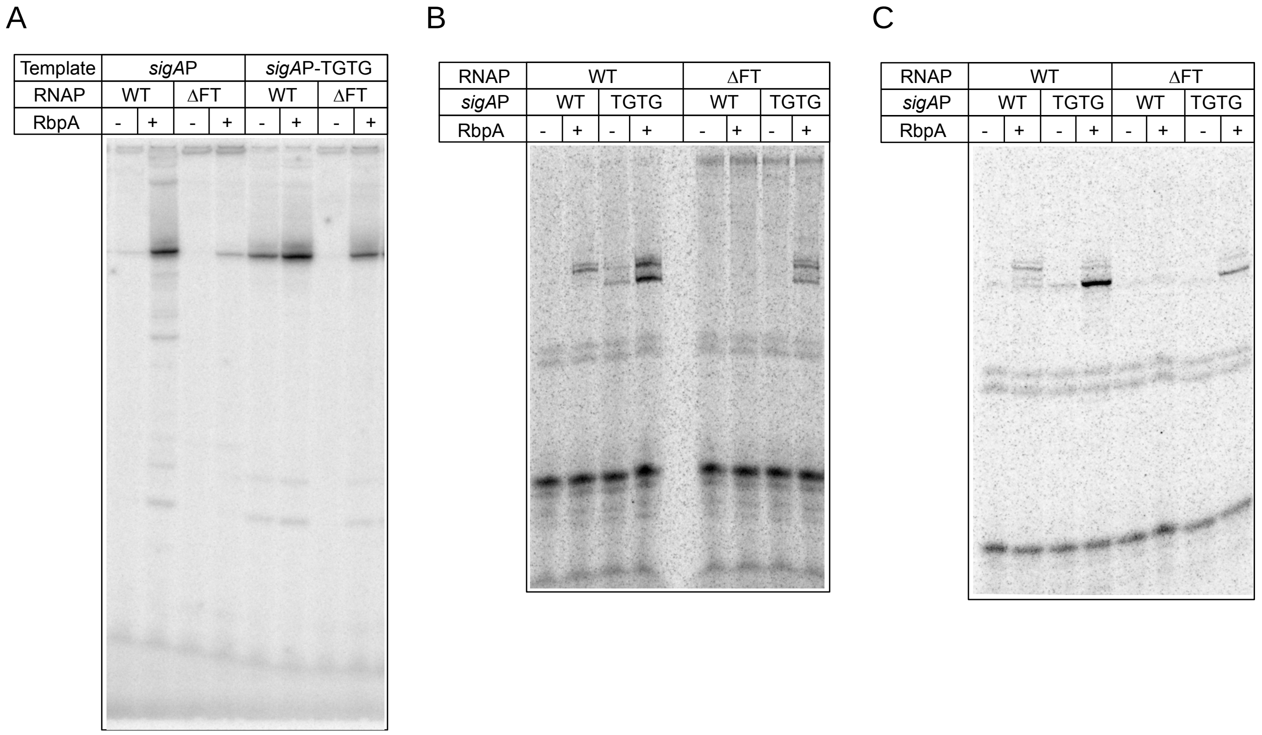

Figure S9. Transcriptional activity of the *Mtb* RNAP. Effect of β-FT deletion (ΔFT) on run-off transcription from the *sigA*P and extended -10 type *sigA*P-TGTG promoters. The [^{32}P]-RNA products (A) and [^{33}P]-RNA products (B,C) were resolved on 24% PAGE/7M Urea. Three replicates are shown.

Supplementary Movie 1. RNAP conformational dynamics revealed by 3DVA. Reconstruction of a series of intermediate maps over each 3DVA component. Component 0: clamp core swiveling-closing. Component 1: σR4 swinging. Component 2: clamp head closing.

References

Fenton MS, Lee SJ & Gralla JD (2000) Escherichia coli promoter opening and -10 recognition: mutational analysis of sigma70. *EMBO J* 19: 1130–7

Schroeder L a., Karpen ME & DeHaseth PL (2008) Threonine 429 of Escherichia coli σ70 is a Key Participant in Promoter DNA Melting by RNA Polymerase. *J Mol Biol* 376: 153–165

Waldburger C, Gardella T, Wong R & Susskind MM (1990) Changes in conserved region 2 of Escherichia coli sigma 70 affecting promoter recognition. *J Mol Biol* 215: 267–276

Waldburger C & Susskind MM (1994) Probing the informational content of Escherichia coli sigma 70 region 2.3 by combinatorial cassette mutagenesis. *J Mol Biol* 235: 1489–1500

Zenkin N, Kulbachinskiy A, Yuzenkova Y, Mustaev A, Bass I, Severinov K & Brodolin K (2007) Region 1.2 of the RNA polymerase sigma subunit controls recognition of the -10 promoter element. *EMBO J* 26: 955–964
